# Optimization of Parameters of Block-Shaped Support Tooth Structure Using Orthogonal Experimental Design in Laser Powder Bed Fusion

**DOI:** 10.3390/ma19081480

**Published:** 2026-04-08

**Authors:** Zhongli Li, Guosheng Fei, Daijian Wu, Xiaoci Chen, Yingyan Yu, Zuofa Liu, Jiansheng Zhang, Jie Zhou

**Affiliations:** 1 Sichuan Provincial Engineering Research Center of Advanced Manufacturing Technology of Ramjet Engines, Sichuan Polytechnic University, Deyang 618000, Chinazhangjiansheng@cqu.edu.cn (J.Z.); zhoujie@cqu.edu.cn (J.Z.); 2 Chongqing Key Laboratory of Advanced Mold Intelligent Manufacturing, College of Materials Science and Engineering, Chongqing University, Chongqing 400044, China; 3 Chongqing Jiepin Technology Co., Ltd., Chongqing 401329, China

**Keywords:** L-PBF, Taguchi experimental design, additive manufacturing, support structure, parameter optimization

## Abstract

To address the challenges associated with laser powder bed fusion (LPBF) of overhanging structures—namely warping deformation, powder adhesion, and inadequate forming accuracy—this study investigates the optimization of the support–part contact interface using Inconel 625 alloy. The objective is to achieve high-quality part formation with minimal support structures. A Taguchi experimental design was employed to systematically evaluate the effects of key block support parameters—tooth height, tooth top length, tooth base length, and tooth base spacing—on the forming performance of overhanging structures, with forming accuracy and support removability as the optimization targets. The results reveal that tooth top length significantly influences both the forming accuracy of overhanging specimens and the ease of support removal. Specifically, an increase in tooth top length leads to a rapid reduction in specimen deformation, but simultaneously increases the difficulty of support removal. When the tooth top length was set to 0.1 mm, all overhanging specimens failed to form successfully. Tooth base length also plays a critical role in support removability, with removal difficulty initially decreasing and then stabilizing as the tooth base length increases. Based on the trade-off between forming quality and support removability, the optimal parameter combination was identified as: tooth height of 0.4 mm, tooth top length of 0.7 mm, tooth base length of 1.0 mm, and tooth base spacing of 0.3 mm. A validation experiment conducted using this optimized configuration demonstrated good forming accuracy in the support contact area, with a deformation value of −0.208 mm, confirming the effectiveness and reliability of the proposed parameters. This study not only provides a theoretical foundation for the optimal design of block supports in LPBF but also offers experimental data and practical guidance for selecting support parameters in the fabrication of overhanging structures.

## 1. Introduction

As one of the core driving forces behind the paradigm shift of Industry 4.0, Additive Manufacturing (AM) has experienced rapid development over the past decade, owing to its unique advantages in fabricating complex structures [1,2]. Among various metal AM technologies, laser powder bed fusion (LPBF) stands out as the most widely adopted technique, characterized by high manufacturing freedom, integrated forming capability, and superior production efficiency. These attributes make it particularly suitable for manufacturing precision components with intricate geometries and stringent performance requirements in aerospace, mold, medical, and other high-tech industries [3,4]. However, when forming structures with large overhang angles using the LPBF process, parts are susceptible to warping, deformation, and even build failure due to severe temperature gradients and thermal stress accumulation. This challenge has emerged as a critical bottleneck restricting the expansion of LPBF into the realm of high-end precision manufacturing [5,6].

To address these challenges, support structures have become indispensable auxiliary components in the LPBF process, with their design rationality directly determining both the forming quality and manufacturing economy of parts [7,8]. The core functions of support structures are primarily manifested in three aspects: providing reliable mechanical support for overhanging regions, acting as heat dissipation paths to optimize temperature field distribution, and ensuring the stability of the layer-by-layer manufacturing process. The fundamental objective of support structure design is to minimize material consumption, reduce post-processing difficulty, and shorten manufacturing cycles while guaranteeing part quality. Among various support configurations, block-type supports are widely employed for supporting overhanging areas of large-volume parts due to their simple structure, high support reliability, and favorable process adaptability [9,10]. Their performance directly dictates the forming accuracy, residual stress level, and post-processing efficiency of overhanging structures, thus becoming one of the core directions in support structure optimization research. As the key design elements of block supports, the optimal configuration of tooth profile structural parameters plays a decisive role in enhancing support performance.

To date, numerous scholars have conducted extensive research on the design and optimization of block support structures, establishing a solid foundation for their engineering applications. Su et al. [11] systematically reviewed the design, optimization, and removal methods of AM support structures, emphasizing that support structures are crucial for preventing forming failure and part warping. They discussed innovative removal technologies including mechanical removal, chemical dissolution, and dissolvable supports, providing comprehensive guidance for support structure optimization and indirectly highlighting the impact of tooth profile parameters on support removability. Calignano [12] designed block supports using Materialise Magics software and investigated the influence of rectangular geometric parameters on the deformation and bottom surface quality of overhanging samples based on the gap model between overhanging structures and solid blocks, aiming to reduce warping deformation and improve support removal convenience—this work laid the groundwork for tooth profile parameter optimization. Nie et al. [5] demonstrated that for the Inconel 718 nickel-based superalloy, block supports exhibit superior performance compared to conical and rhombic lattice supports in inhibiting deformation and reducing residual stress, further validating the advantages of block supports in processing high-performance materials and providing a reference for investigating material adaptability in tooth profile parameter optimization.

Huang et al. [13] proposed a block–grid–tree composite support structure for titanium alloy parts, which demonstrated significant advantages in reducing support volume and controlling warping, thereby enriching the structural variants of block supports. However, this study did not involve in-depth refinement and optimization of tooth profile parameters. Khobzi et al. [14] investigated the influence of two types of square solid support structures on the thermal field and deformation during LPBF forming, revealing that the total contact area of the support top and the base area play a pivotal role in thermal field distribution. This finding established the intrinsic correlation between the macro-structural parameters of block supports and forming performance, providing theoretical support for optimizing tooth profile parameters such as tooth top length and tooth base spacing.

Regarding parameter optimization and performance characterization of block supports, substantial research efforts have been undertaken, yet notable deficiencies remain in the systematic optimization of tooth profile parameters, and the application of Taguchi experimental methods has not been fully established. Dimopoulos et al. [15] employed experimental design and multi-response optimization methods to systematically analyze the influence of tooth height, tooth top length, grid spacing, laser speed, and overhang angle on the volume, removal difficulty, and warping deformation of Ti6Al4V support structures. They identified optimal parameter combinations under various overhang angles, marking the first incorporation of tooth profile-related parameters into the block support optimization framework. However, this study paid insufficient attention to the interactions among tooth profile geometric parameters, nor did it thoroughly investigate their influence mechanisms on the forming accuracy of cantilever structures, and the Taguchi method was not adopted for multi-objective optimization. Zhang et al. [16] focused on Ti-6Al-4V block supports, systematically exploring the effects of laser power, scanning speed, and layer thickness on their forming quality and mechanical properties. While determining optimal process parameter combinations and providing a basis for block support quality control, this research did not address the optimization of tooth profile geometric parameters.

Furthermore, critical performance attributes of support structures—including heat conduction functionality, interfacial mechanical properties, and removability—are intimately related to tooth profile geometry, further underscoring the necessity of tooth profile parameter optimization. Craeghs et al. [17,18] demonstrated that support structures promote molten pool heat dissipation, reducing the average molten pool size and thermal stress, with tooth profile design directly affecting the rationality of heat dissipation paths and heat conduction efficiency, thereby influencing part forming accuracy. Lindecke et al. [19] investigated the mechanical properties of the support–part interface, revealing that interface strength is substantially lower than that of bulk materials and is significantly influenced by factors including tooth profile geometry. This interface strength directly determines both the reliability and removability of supports, providing clear direction for tooth profile parameter optimization. Yue et al. [20] studied the influence of support structures and build orientation on the tensile properties of AlSi10Mg overhanging structures. Their results showed that support structures effectively reduce the initial layer molten pool temperature by 5.3%, with mechanical properties following the order: 45° specimen above solid > 45° specimen with support > 45° specimen without support. These findings confirmed that rational support parameter design is the key to minimizing forming defects in overhanging areas and improving mechanical properties.

Cao et al. [21] conducted a comparative study revealing that 316L stainless steel block supports exhibit superior machinability compared to conical supports during mechanical milling removal. They attributed this difference in removability primarily to variations in tooth profile geometric parameters, further validating the importance of tooth profile parameter optimization in enhancing post-processing efficiency. Ameen et al. [22,23] optimized support parameters using response surface methodology and multi-objective genetic algorithms, advancing the LPBF process toward higher efficiency and quality. However, these studies did not systematically investigate the interactions among tooth profile geometric parameters of block supports, nor did they integrate Taguchi experimental methods to achieve efficient parameter optimization. Gülcan et al. [24] adopted a multi-response optimization approach to systematically investigate the influence of tooth profile support geometric parameters on overhanging structure forming quality. Through experimental design and 3D printing, they identified optimal parameter combinations for 30° overhang angles, establishing that tooth height is the key factor affecting support volume and dimensional accuracy, while tooth base length dominates surface roughness and microhardness. Nevertheless, this study did not address tooth profile geometric parameter optimization for 0° overhang angles, nor did it employ the Taguchi method for parameter optimization, limiting its ability to achieve efficient multi-objective trade-offs.

Hassanin et al. [25] optimized LPBF process parameters using response surface methodology, improving the surface roughness and density of Ti6Al4V components, thereby providing a reference for subsequent matching optimization of tooth profile parameters and process parameters. Markovits et al. [26] investigated the influence of support structure parameters and laser power on connection strength through torsion tests, identifying the effects of grid spacing and tooth top length on connection strength, and conducted multi-objective optimization using the Taguchi method. While this work highlighted both the importance of tooth profile-related parameters and the advantages of the Taguchi experimental method, it did not establish a systematic tooth profile parameter optimization framework, nor did it focus specifically on in-depth investigation of block support tooth profile parameters.

In summary, among numerous support configurations, block supports have emerged as the preferred choice in many high-performance material manufacturing scenarios due to their balanced performance in structural reliability, thermal management efficiency, and process universality. Their performance largely depends on the design of tooth profile geometric parameters—including tooth height, tooth top length, tooth base length, and tooth base spacing—and their matching with process parameters. These parameters collectively determine the bonding strength, stress distribution, heat dissipation efficiency, and final separability of the support–part contact interface [27,28], representing critical factors affecting cantilever structure forming accuracy and support removability. However, existing studies predominantly focus on the individual effects of a limited set of tooth profile parameters, lacking a systematic understanding of the complex interaction mechanisms among these geometric parameters. In particular, it remains unclear how these parameters influence macro-performance through their effects on micro-morphology and interfacial metallurgical bonding [29,30], and quantitative research on the impact of tooth profile parameters on cantilever structure forming accuracy remains scarce. Furthermore, although progress has been made in optimizing block supports for different material systems (such as Inconel 718 and Ti6Al4V), the universality and transferability of tooth profile parameter optimization conclusions require further validation.

In response to the aforementioned research gaps, this study focuses on the optimization of block support tooth profile structural parameters, employing the Taguchi experimental design method to systematically investigate the effects of tooth height, tooth top length, tooth base length, and tooth base spacing on the forming quality of cantilever structures and the difficulty of support removal. Through experimental validation, the influence mechanisms of each parameter on cantilever structure forming accuracy and support removability are quantified, leading to the identification of optimal tooth profile parameter combinations. This study not only provides a theoretical foundation for the optimal design of block supports in LPBF processes but also offers experimental data support and practical references for designing support parameters for components with cantilever structure characteristics fabricated by laser powder bed fusion.

## 2. Materials and Methods

### 2.1. Experimental Materials and Equipment

**Figure 1:**
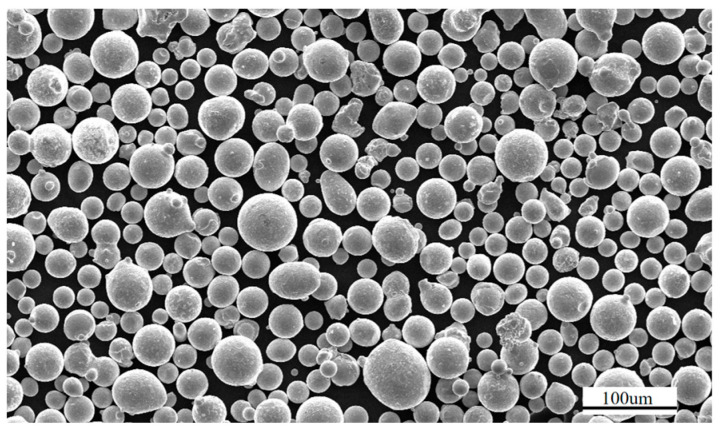
Inconel625 powder particle size morphology.

The experimental raw material used was Inconel625 nickel-based high-temperature alloy powder, with a chemical composition shown in Table 1 [31]. The particle size distribution was 15–54 um, with particle size distributions of D (10): 21.53 um, D (50): 33.162 um, and D (90): 51.37 um, the particle size and morphology of GH3625 powder are shown in Figure 1. The loose density of the powder is 4780 kg·m^−3^. The material has a solid density of 8240 kg·m^−3^, a Poisson’s ratio of 0.3, and a melting point of 1298 °C.

The preparation of experimental specimens in this study was carried out using a Laser Powder Bed Melting forming equipment with the model LiM-X260A. This device has high beam quality, high-precision positioning, and wide-range process parameter adjustment capabilities, which can meet the accuracy and performance requirements of experimental piece forming. Its main technical parameters are shown in Table 2.

In this study, a GOM SCAN composite 3D scanner was employed for three-dimensional data acquisition and accuracy inspection. The scanner operates in fixed binocular mode and offers high-resolution data capture capabilities. With a detection resolution of 0.005 mm in blue light scanning mode, it can precisely capture the geometric features and minor deformations on the specimen surface. This provides reliable data support for subsequent dimensional accuracy analysis.

### 2.2. Model Construction

**Figure 2:**
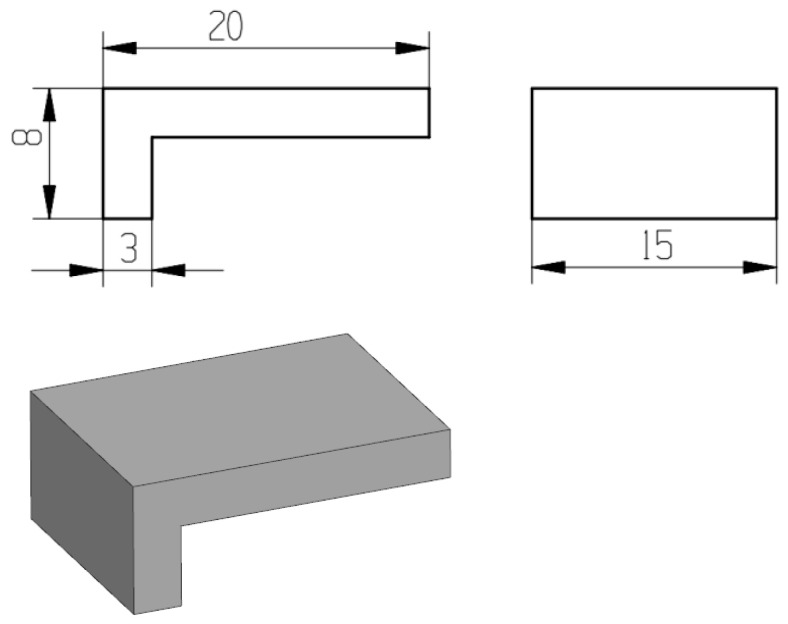
Structure and dimensions of the experimental piece (unit: mm).

To investigate the influence mechanism of block support tooth parameters on the forming accuracy of cantilever structures, a three-dimensional model of the test specimen was first established using NX 12.0 CAD software. The cantilever specimen has dimensions of 20 mm × 15 mm × 8 mm (length × width × height), as illustrated in Figure 2.

**Figure 3:**
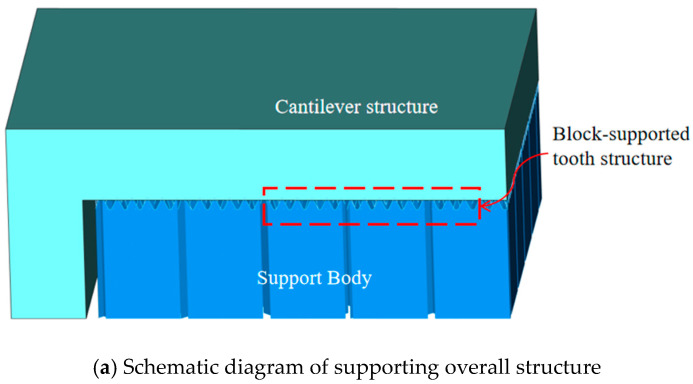

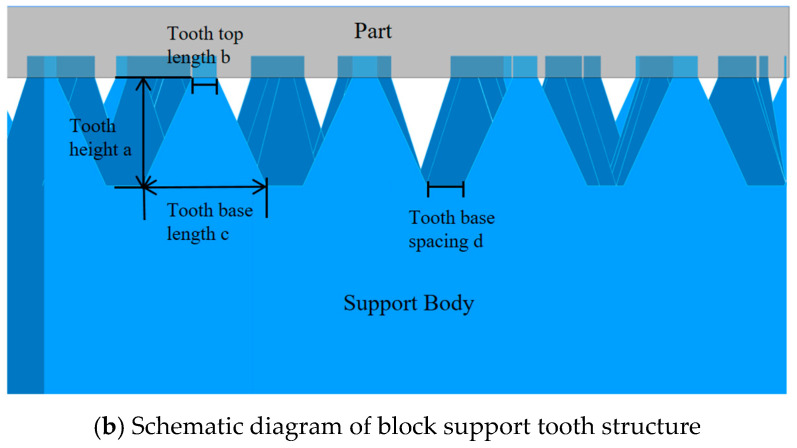
Schematic diagram of block support for suspended structural components.

After modeling, the suspension arm test specimen was imported into Magics 24.0 software. Block supports were subsequently generated on the structurally critical overhang surfaces—areas particularly susceptible to forming defects. The key geometric parameters of the support teeth are defined as follows: tooth height (a), tooth top length (b), tooth base length (c), and tooth base spacing (d). The contact interface between the block support and the cantilever specimen, the specific values of these parameters, and the overall support layout are presented in Figure 3.

### 2.3. Taguchi Experimental Design

The tooth structure of the block support is in direct contact with the cantilever component surface, exerting a significant influence on the forming quality of the component. The geometric parameters of the support structure govern the heat transfer path and heat accumulation behavior in the overhang region. Specifically, the effective contact area between the support and the overhang surface directly determines the interfacial thermal conductivity, thereby regulating the melt pool cooling rate and thermal gradient distribution. In general, a larger contact area facilitates rapid heat dissipation and reduces thermal stress levels; however, excessively strong interfacial bonding simultaneously increases the difficulty of support removal. Therefore, understanding the coupling relationship among heat transfer, melt pool behavior, and interfacial bonding strength is essential for elucidating the variations in deformation and support removability.

Based on the aforementioned physical mechanisms, this study employed the Taguchi experimental design method to systematically investigate the effects of block support tooth parameters on the forming performance of cantilever components. Four tooth parameters—tooth height, tooth top length, tooth base length, and tooth base spacing—were selected, each at four levels, to construct an L16 (45) orthogonal array. The forming deformation of cantilever specimens and support removability were adopted as the primary evaluation indicators to reveal the main effects and underlying mechanisms of each tooth parameter. The specimens are designated as C-n. The orthogonal array header design, factor-level table, and complete experimental scheme are presented in Table 3, Table 4, and Table 5, respectively.

It is worth noting that while the Z-offset is a critical process parameter influencing build quality in laser powder bed fusion, this study specifically focuses on the effect of the support structure’s geometric characteristics on the deformation of cantilever components. Therefore, in the Taguchi design, only the tooth width, tooth height, and tooth angle were selected as investigative factors, with the Z-offset held constant at 0.08 mm to isolate the effects of the geometric variables.

### 2.4. Process Parameters

This study focuses on Inconel625 powder alloy and uses laser powder bed melting (LPBF) to prepare cantilever structural components. To ensure the forming accuracy of cantilever specimens, differentiated process parameter design was adopted for the solid area and support area during the laser powder bed melting forming process. The specific printing process parameters for cantilever components and block supports are shown in Table 6.

### 2.5. Three-Dimensional Scanning and Detection Technology

This study used GOM SCAN 3D laser scanner to carry out model data scanning and acquisition work. This device has high-precision point cloud acquisition capability (scanning accuracy ≤ 0.01 mm), which can effectively capture the micro geometric features of component surfaces and ensure the reliability of raw data. During the data collection process, the full domain 3D point cloud data of the experimental piece is obtained through non-contact scanning, and then the triangular surface reconstruction algorithm is used to encapsulate the point cloud data into stl format files, providing data support for subsequent 3D dimensional accuracy detection.

In the 3D inspection stage, based on the principle of geometric accuracy analysis, the Best Fit Alignment method was employed to perform 3D alignment between the scanned model and the original CAD design model. This method iteratively optimizes the overall geometric deviation between the two models using the least squares algorithm, eliminating the spatial offset between the measurement coordinate system and the design coordinate system, thereby ensuring the objectivity and accuracy of the alignment results.

Based on the deformation characteristics of the cantilever components, measurement points were selected at the contour edge where the tooth structure interfaces with the lower surface of the overhang. Deformation in this region directly reflects the response of the tooth structure to thermal stress. Due to insufficient support strength, the contour edge exhibits a negative deviation (material deficit) as a result of warping deformation. Therefore, absolute values were adopted in the subsequent Taguchi analysis to quantitatively characterize the degree of deformation.

During point selection, measurements were not taken arbitrarily. Instead, 3 to 5 characteristic points were selected from regions exhibiting relatively large contour deformation (i.e., areas with deeper coloration in the chromatogram). The average of these measurements was used as the maximum deformation index for the cantilever component, ensuring that the measurement results objectively reflect the actual deformation characteristics of the component.

## 3. Results and Discussion

### 3.1. Orthogonal Experimental Results

**Figure 4:**
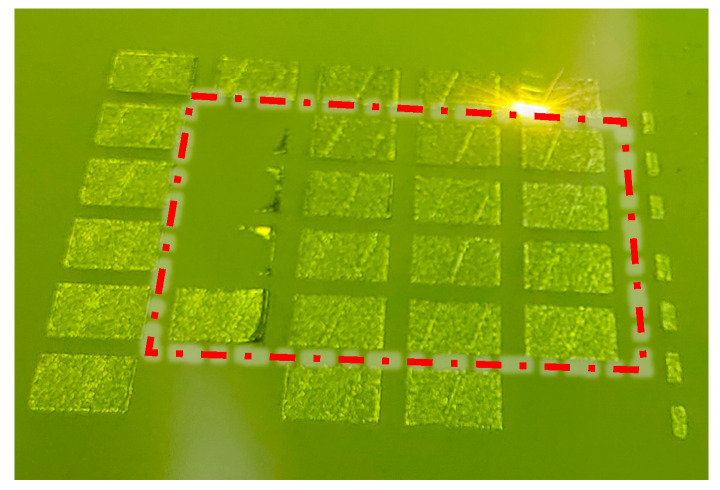
During the forming experiment.

**Figure 5:**
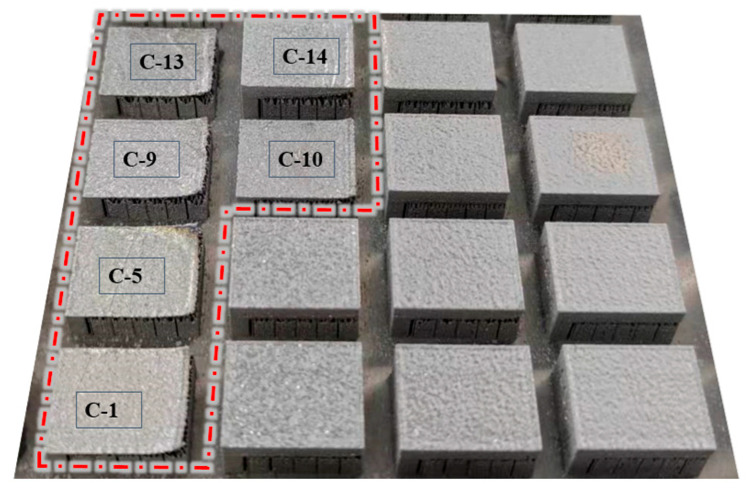
Forming photos.

The laser powder bed fusion (LPBF) forming process and the corresponding as-built samples are presented in Figure 4 and Figure 5, respectively. In Figure 4, the red box highlights the printing status of the 16 groups of samples during fabrication. In Figure 5, red circles indicate samples that exhibited forming abnormalities during the process, specifically samples C-1, C-5, C-9, C-10, C-13, and C-14. Among these, five samples (C-1, C-5, C-9, C-10, and C-13) experienced severe warping deformation of the overhanging surface during printing. This deformation not only significantly compromised the powder spreading quality but also posed a risk of scraper damage. Based on real-time observation, the printing of these five samples was deliberately terminated. For sample C-14, notable warping occurred at the initial stage of overhanging surface formation, with a tendency to progressively deteriorate and interfere with the recoating process. However, as the number of printed layers increased, the adverse effect of warping on powder spreading gradually diminished, and the sample was ultimately completed without further incident. The remaining ten samples exhibited no significant anomalies throughout the entire forming process. These results provide direct experimental evidence that variations in the tooth profile parameters of block supports significantly influence the forming quality of cantilever components.

In the experiment on block-type support tooth parameters, the printing failures of cantilever components were attributed to insufficient bonding strength at the interface between the solid part and the supports. This issue primarily stemmed from inappropriate combinations of tooth parameters, which resulted in a limited contact area. Under high-energy laser irradiation, the molten metal powder failed to dissipate heat to the substrate in a timely manner. The consequent heat accumulation induced warping deformation of the component. Furthermore, the selected support tooth parameters could not provide adequate restraining force to counteract the upward lifting of the overhanging surface, leading to severe distortion and eventual print failure.

**Figure 6:**
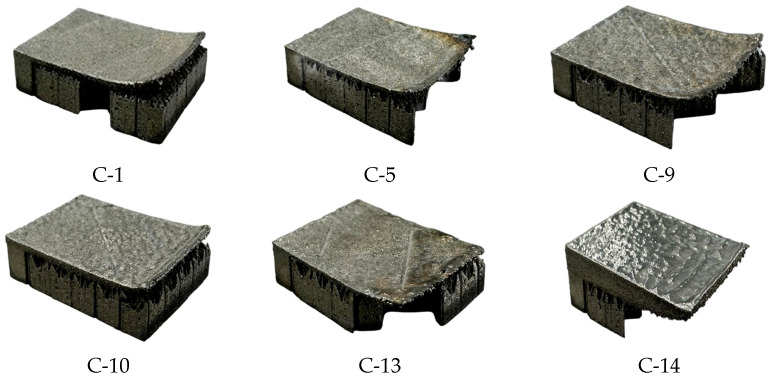
Photographs and identification numbers of five sets of failed printed samples.

Photographs of the specimens that exhibited anomalies during the printing process are presented in Figure 6. Among these, five specimens (Nos. C-1, C-5, C-9, C-10, and C-13) experienced severe warping deformation and were proactively terminated following comprehensive assessment; accordingly, they are categorized as “printing failure” samples. In contrast, although specimen No. C-14 also underwent significant deformation, it successfully completed the forming process.

In the orthogonal experimental design employed in this study for parameter optimization, the deformation of overhang structures is characterized as a “smaller-the-better” characteristic. For certain specimens, the excessive deformation during fabrication led to premature termination of the printing process, rendering the acquisition of valid deformation measurements impossible. This outcome inherently indicates that, under the current process conditions, the corresponding parameter combinations are “unacceptable,” with deformation values that should be substantially larger than those of all successfully fabricated specimens.

To enable quantitative analysis of the orthogonal experimental results, a reasonable imputation of these missing data points was necessary. The imputation procedure was as follows: First, the maximum deformation value among all successfully fabricated specimens was determined and denoted as $Y_{\max}$. Based on the rationale that the deformation of “failed” specimens should significantly exceed that of successful ones, the five missing data points were assigned a value slightly greater than $Y_{\max}$. The assignment criterion can be expressed as:

$$Y_{mis}=Y_{\max}\times (1+\delta )$$

where δ is recommended to range between 5% and 10%.

In this experiment, the maximum measured deformation of the successfully fabricated samples was approximately 1.9 mm. Accordingly, the deformation values for the five groups of cantilever components that failed to form were uniformly assigned as 2 mm. Subsequent analysis revealed that although this assignment had a certain influence on the deformation range in the range analysis of the Taguchi method, it did not introduce any substantive contradiction in the analysis of variance results, nor did it alter the main conclusions of the study. This confirms the validity of the assigned value, indicating that it effectively reflects the influence of tooth geometry parameters on the deformation behavior of cantilever structures.

It should be noted that the maximum deformation of all cantilever components occurred at the contour boundary where the lower surface of the overhang contacts the toothed supports. Due to insufficient support strength, warping deformation took place at this boundary, resulting in a negative deviation from the designed model. To facilitate calculation and statistical analysis, the absolute values of the deformation were adopted for data processing.

To ensure the objectivity and comparability of the experimental results, all support removal operations were carried out by the same operator under consistent laboratory conditions using an identical set of tools. This approach helped minimize the influence of subjective variability among operators on the evaluation outcomes.

In this experiment, the operator also applied the same tools to the unsupported regions, with an emphasis on assessing both the difficulty of support removal and the extent of residual support remaining. Based on the complexity of post-processing removal, the following four evaluation grades were defined:

Grade 1 (Easy removal): Supports can be removed effortlessly, either without tools or with minimal manual assistance, leaving little to no residue on the support surface.

Grade 2 (Moderate removal): Supports can be removed using tools, but noticeable residue remains on the surface after removal.

Grade 3 (Difficult removal): Supports require the use of additional tools (excluding machine tools) for removal.

Grade 4 (Extremely difficult removal): Supports cannot be removed manually and must be eliminated through machining processes.

The results of the dimensional accuracy tests and the corresponding support removal difficulty for the cantilever structural components are presented in Table 7.

### 3.2. The Influence of Tooth Parameters on the Forming Accuracy of Cantilever Components

Taking the deformation of the contact surface supported by the suspended bridge structure as the optimization objective, orthogonal experimental analysis was conducted on the above results to obtain the range analysis (Table 8) and variance analysis (Table 9) of the deformation of the suspended bridge structure.

According to the range analysis presented in Table 7, the influence of tooth parameters on the deformation of the test specimens, in descending order of significance, is as follows: tooth top length (b) > tooth base spacing (d) > tooth base length (c) > tooth height (a). The experimental results reveal that all specimens with a tooth top length of 0.1 mm failed to form successfully, further confirming that tooth top length (b) exerts a significant influence on specimen deformation.

This failure can be attributed to the substantial reduction in interfacial thermal conductivity resulting from the excessively small contact area. At a tooth top length of 0.1 mm, the effective contact area between the support and the fabricated part is extremely limited, leading to severely inadequate heat transfer at the interface. Under high-energy laser irradiation, the heat generated in the melt pool cannot be efficiently dissipated to the substrate via the support structure. This results in intensified local heat accumulation, expansion of the melt pool, and prolonged residence time at elevated temperatures. As illustrated in Figure 5, the consequent excessive thermal gradient induces significant thermal stress. When this stress exceeds the material’s yield strength, irreversible warping deformation occurs, ultimately leading to forming failure.

The analysis of variance (ANOVA) results in Table 8 assess the significance of each factor using *p*-values. Typically, a *p*-value below a predetermined significance threshold (e.g., 0.05) leads to the rejection of the null hypothesis, indicating that the factor exerts a statistically significant influence on the experimental results. As shown in the table, factor b (tooth top length) yields an F-value of 22.66 and a *p*-value below 0.05, confirming its significant statistical effect. This implies with over 95% confidence that the observed inter-group differences are not attributable to random error. The strong agreement between the range analysis and ANOVA further substantiates the reliability of the experimental data and the robustness of the conclusions.

**Figure 7:**
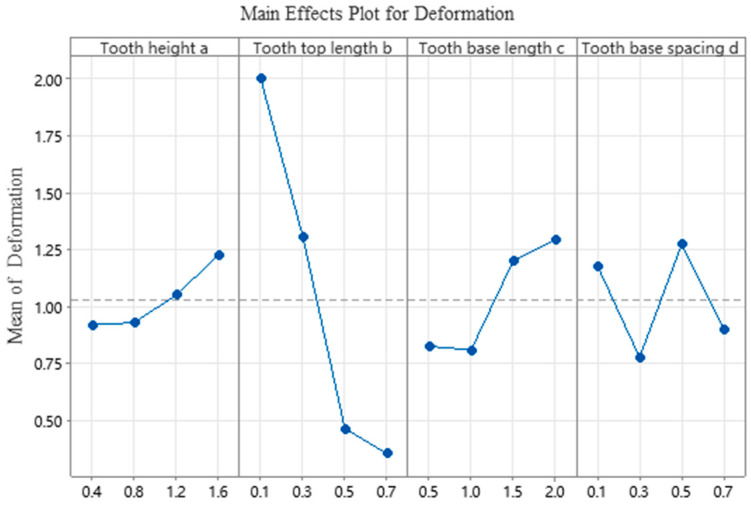
The influence trend of various factors of tooth parameters on deformation.

The influence trend of various factors of tooth parameters on the deformation of cantilever structural components is shown in Figure 7. The results show that the deformation increases with the increase in tooth height a and tooth base length c; as the length of the tooth tip b increases, the amount of deformation decreases. According to the principle of minimizing deformation, the optimal coefficient combination is derived as A1B4C2D2, which means that the tooth height a is 0.4 mm, the tooth tip length b is 0.7 mm, the tooth base length c is 1.0 mm, and the tooth base spacing d is 0.3 mm.

### 3.3. Analysis of the Influence of Tooth Parameters on the Difficulty of Support Removal

Based on the definition of the difficulty level of support removal mentioned earlier, as shown in Table 6, it can be concluded that the overall removal of block support parameters is good and can be achieved without the need for machine tool processing. However, after removal, there is a significant amount of residual slag on the surface, resulting in poor surface quality.

The average and range analysis of the difficulty of removing different parameter factors of tooth structure are shown in Table 10. The results show that the length b of the tooth top has the greatest impact on the difficulty of removing support, followed by the length c of the tooth base, the height a of the tooth, and the spacing d between the tooth base. It can be seen that the length b of the tooth top is the main factor affecting the difficulty of removing the support.

According to the analysis of variance (ANOVA) results presented in Table 11 for the difficulty of support removal, the *p*-value serves as an indicator of factor significance. Typically, if a *p*-value falls below a predetermined significance threshold (e.g., 0.05), the null hypothesis is rejected, signifying that the corresponding factor exerts a statistically significant influence on the experimental outcome. As shown in the table, both tooth top length (b) and tooth base length (c) yield *p*-values below 0.05, with respective F-values of 19.67 (*p* = 0.018) and 14.33 (*p* = 0.028). These results indicate that both factors possess significant statistical effects, confirming with over 95% confidence that the observed inter-group differences are not attributable to random error. The strong agreement between the ANOVA and range analysis further substantiates the reliability of the experimental data and the robustness of the conclusions drawn.

**Figure 8:**
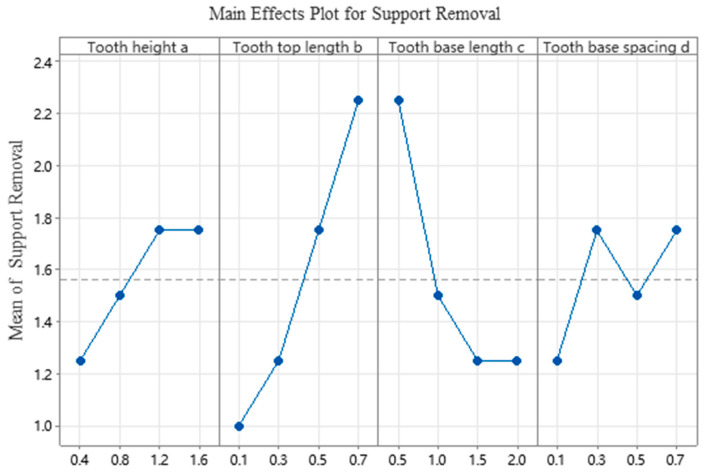
Effect curve of various tooth parameters on the difficulty of support removal.

Figure 8 presents the main effects plot of each tooth parameter on support removability. The results show that tooth top length significantly influences the ease of support removal: as tooth top length increases, support removal becomes progressively more difficult. This trend can be attributed to the evolution of interfacial bonding strength. At smaller tooth top lengths, the effective contact area between the support and the fabricated part is limited, resulting in weak interfacial bonding and consequently, easier support removal. As tooth top length increases, the effective contact area expands, enhancing interfacial heat transfer and promoting stronger metallurgical bonding. Accordingly, the difficulty of support removal initially increases sharply and then stabilizes. The identification of this critical threshold reveals a saturation effect in the regulation of interfacial bonding by support geometric parameters, offering a theoretical basis for parameter optimization. Similar thermal–mechanical coupling mechanisms have been reported in LPBF forming studies [27,28].

From the perspective of support removability alone, the optimal parameter combination is A_1_B_1_C_3_(D_4_)D_1_, i.e., tooth height of 0.4 mm, tooth top length of 0.1 mm, tooth base length of 1.5 mm (or 2.0 mm), and tooth base spacing of 0.1 mm.

### 3.4. Experimental Verification

According to Section 3.1, Section 3.2 and Section 3.3, it can be seen that the tooth top length b in the tooth structure parameters has the greatest impact on the forming accuracy of the suspension structure and the difficulty of removing the support. It can be seen that the larger the length b at the top of the tooth, the smaller the deformation of the cantilever structure, and the higher the forming accuracy. However, the larger the length b at the top of the tooth, the more difficult it is to remove the support. There is a negative correlation between forming accuracy and support removal difficulty, which cannot be met simultaneously.

**Figure 9:**
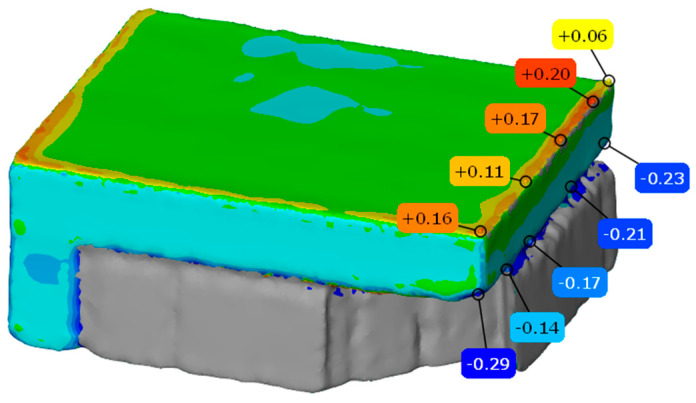
Three-dimensional accuracy assessment results for the cantilever component.

In practical process design, the principle of prioritizing forming accuracy should be considered, and the ease of support removal should be taken into account while ensuring forming quality. Therefore, the optimal combination of tooth structure parameters A1B4C2D2 was selected for the orthogonal experiment to verify the process. The developed cantilever component was subjected to three-dimensional scanning inspection, and the three-dimensional inspection results are shown in Figure 9.

The 3D inspection results show that the upper surface of the cantilever component presents a trend of high to medium flatness at the four edges, and the overall deviation in the middle part of the upper surface is not large, ranging from −0.1 to 0.1. The maximum deviation at the four edges is +0.20 mm; the maximum deformation at the contact edge between the support and the lower surface of the cantilever is −0.29 mm; and the average deformation is −0.208 mm. Within the process window, the effectiveness of this combination parameter is verified by considering both forming accuracy and support removal difficulty.

## 4. Conclusions

Block support is a commonly used support structure in laser selective melting technology. To investigate the influence of block-shaped support tooth parameters on the forming accuracy of cantilever components, an orthogonal experimental method was adopted in this study. The effects of block-shaped support structure parameters on both the forming accuracy and the difficulty of support removal were systematically analyzed. The main findings are as follows:

(1) The influence of tooth structure parameters on the forming accuracy of the overhanging structure, in descending order of significance, is: tooth top length (b) > tooth base spacing (d) > tooth base length (c) > tooth height (a).

(2) Regarding the difficulty of support removal, tooth top length (b) exhibits the most significant effect, followed by tooth base length (c), tooth height (a), and tooth base spacing (d).

(3) In practical application, priority should be given to forming accuracy when selecting tooth structure parameters. Accordingly, a parameter combination of A1B4C2D2 was validated through orthogonal experimentation. The results show that the maximum deformation at the support contact surface of the cantilever component was −0.29 mm, with an average deformation of −0.208 mm, confirming the effectiveness of this parameter combination.

The findings of this study demonstrate that the optimized block-shaped support tooth parameters can be effectively applied to the design of lower surface support teeth for single cantilever structures fabricated from the same material. Furthermore, these results offer essential experimental data support and a valuable reference for the design of support tooth parameters for similar cantilever structural features in laser powder bed fusion.

## Figures and Tables

**Table 1:** Chemical composition of GH3625 nickel-based high-temperature alloy powder [31].

Si	Mn	*p*	S	Cr	Cu	Mo	Ti	Nb	Al	Co	Fe	Ni
0.036	<0.005	<0.005	0.0004	21.8	0.0022	9.02	0.11	3.72	0.14	0.0011	1.98	Bal.

**Table 2:** Technical parameters of laser selective melting equipment.

Technical Indicators	Parameter
Maximum power/w	500
Laser wavelength/nm	1064
Beam quality factor	M^2^ ≤ 1.1
Positioning accuracy/mm	±0.005
Scanning speed/(mm/s)	100~7000
Spot diameter/um	80~100
Layered thickness/um	20~80
Forming space/mm	260 × 260 × 430

**Table 3:** Orthogonal experiment header design.

Factors	A	B	C	D	E
4	Tooth height	Tooth top length	Tooth base length	Tooth base spacing	Error column

**Table 4:** Factor-level table (unit: mm).

Level/Factors	a	b	c	d
Level 1	0.4	0.1	0.5	0.1
Level 2	0.8	0.3	1	0.3
Level 3	1.2	0.5	1.5	0.5
Level 4	1.6	0.7	2	0.7

**Table 5:** Orthogonal experiment table (unit: mm).

Number/Factor	a	b	c	d	Error Column
C-1	0.4	0.1	0.5	0.1	1
C-2	0.4	0.3	1	0.3	2
C-3	0.4	0.5	1.5	0.5	3
C-4	0.4	0.7	2	0.7	4
C-5	0.8	0.1	1	0.5	4
C-6	0.8	0.3	0.5	0.7	3
C-7	0.8	0.5	2	0.1	2
C-8	0.8	0.7	1.5	0.3	1
C-9	1.2	0.1	1.5	0.7	2
C-10	1.2	0.3	2	0.5	1
C-11	1.2	0.5	0.5	0.3	4
C-12	1.2	0.7	1	0.1	3
C-13	1.6	0.1	2	0.3	3
C-14	1.6	0.3	1.5	0.1	4
C-15	1.6	0.5	1	0.7	1
C-16	1.6	0.7	0.5	0.5	2

**Table 6:** Process parameters for cantilever components and support forming.

Category/Parameters	Power	Scanning Speed	Layer Thickness	Hatch Distance
Cantilever components	285 W	960 mm/s	0.04 mm	0.12 mm
Support	300 W	1650 mm/s	0.08 mm	0.6 mm

**Table 7:** Experimental results.

Number/Factor	a	b	c	d	Error Column	Deformation/mm	Difficulty in Removing Support
C-1	0.4	0.1	0.5	0.1	1	2	1
C-2	0.4	0.3	1	0.3	2	0.6644	1
C-3	0.4	0.5	1.5	0.5	3	0.5617	1
C-4	0.4	0.7	2	0.7	4	0.4554	2
C-5	0.8	0.1	1	0.5	4	2	1
C-6	0.8	0.3	0.5	0.7	3	0.6678	2
C-7	0.8	0.5	2	0.1	2	0.7153	1
C-8	0.8	0.7	1.5	0.3	1	0.3361	2
C-9	1.2	0.1	1.5	0.7	2	2	1
C-10	1.2	0.3	2	0.5	1	2	1
C-11	1.2	0.5	0.5	0.3	4	0.1075	3
C-12	1.2	0.7	1	0.1	3	0.1000	2
C-13	1.6	0.1	2	0.3	3	2	1
C-14	1.6	0.3	1.5	0.1	4	1.9016	1
C-15	1.6	0.5	1	0.7	1	0.4751	2
C-16	1.6	0.7	0.5	0.5	2	0.5273	3

Note: The deformation data in the table are the average values obtained from 3 to 5 measurement points selected at the region where the edge deformation of the contour is the maximum.

**Table 8:** Analysis of range of experimental deformation.

Level	Tooth Height a	Tooth Top Length b	Tooth Base Length c	Tooth Base Spacing d
1	0.9204	2.0000	0.8257	1.1792
2	0.9298	1.3085	0.8099	0.7770
3	1.0519	0.4649	1.1999	1.2722
4	1.2260	0.3547	1.2927	0.8996
Delta	0.3056	1.6453	0.4828	0.4952
Rank	4	1	3	2

**Table 9:** Deformation variance analysis.

Sources	Degree of Freedom	Adj SS	Adj MS	F Value	*p* Value
a	3	0.2438	0.08125	0.77	0.583
b	3	7.1751	2.39170	22.66	0.015
c	3	0.7522	0.25072	2.38	0.248
d	3	0.6478	0.21592	2.05	0.286
Error	3	0.3166	0.10555		
Total	15	9.1354			

**Table 10:** Analysis of difficulty and range of support removal.

Level	Tooth Height a	Tooth Top Length b	Tooth Base Length c	Tooth Base Spacing d
1	1.250	1.000	2.250	1.250
2	1.500	1.250	1.500	1.750
3	1.750	1.750	1.250	1.500
4	1.750	2.250	1.250	1.750
Delta	0.500	1.250	1.000	0.500
Rank	3	1	2	3

**Table 11:** Variance analysis of difficulty in removing support.

Sources	Degree of Freedom	Adj SS	Adj MS	F Value	*p* Value
a	3	0.6875	0.22917	3.67	0.157
b	3	3.6875	1.22917	19.67	0.018
c	3	2.6875	0.89583	14.33	0.028
d	3	0.6875	0.22917	3.67	0.157
Error	3	0.1875	0.06250		
Total	15	7.9375			

## Data Availability

The original contributions presented in this study are included in the article. Further inquiries can be directed to the corresponding authors.
